# Aspects of oral and general health among a community center for the underserved

**Published:** 2011-05-25

**Authors:** R Sfeatcu, A Dumitrache, L Dumitraşcu, D Lambescu, C Funieru, M Lupuşoru

**Affiliations:** ‘Carol Davila’ University of Medicine and Pharmacy, BucharestRomania

**Keywords:** institutionalized people, chronic diseases, risk factors, medications, treatment needs

## Abstract

**Rationale**: oral health is one of the fundamental steps to general health, well being and a determinant factor for the quality of life.

**Objective** of this cross–sectional study is to assess the oral–systemic health and the treatment needs among the institutionalized people in a homeless center, with high prevalence of co–morbidities and barriers to care.

**Methods and Results**: after getting the informed consent, 51 adults from a community, which is disadvantaged from a socio–economic and medical point of view, were studied: frequently with multiple general diseases treated with medicines that produce oral side effects, a high need of treatment and the lack of a dental office. The subjects were orally examined from a clinical point of view and received a questionnaire with regard to the presence of the health risk factors. General health status and drug treatments of subjects were evaluated based on medical records. Results showed that oral health status of the subjects is precarious, oral hygiene is poor and the subjects are exposed to common risk factors for oral and systemic diseases: tobacco use, alcohol consumption, diet, poor hygiene and history of cancer.

**Discussion**: if their needs are not met, the oral health will be persistently poor and will further deteriorate during their residency, because of increasing care dependency and subsequent lack of adequate oral health care. Despite great achievements in oral and general health of populations, problems still remain in many communities, particularly among underprivileged groups.

## Introduction

Oral health as well as general health qualifies as major public health issues and the greatest burden of oral diseases is on disadvantaged and socially marginalized population. The severe impact in terms of pain and suffering, impairment of function and effect on quality of life must also be considered [1]. The interrelationship between oral and general health is proven by evidence [2] and is particularly pronounced among older and vulnerable people [3]. When exploring common pathways in oral–systemic health it is clear that oral health has a major etiological role in the pathogenesis of many common systemic diseases: oral plaque biofilm is a potential source of systemic inflammation. The conditions for the mostly proven oral–systemic shared etiology are cardiovascular disease, diabetes and certain respiratory diseases (chronic obstructive pulmonary disease). Severe periodontal disease is associated with diabetes. 

Poor oral health can increase the risks of general health. Similarly, systemic diseases and/or the adverse side–effects of their treatments can lead to an increased risk of oral diseases, reduced salivary flow (dry mouth), altered senses of taste and smell, oro–facial pain, gingival overgrowth, alveolar bone resorbtion, mobility of teeth [4]. The high prevalence of multi–medication therapies, especially among elderly may further complicate the impact on oral health [5]. Other relevant issues include high sugar content diets, inadequate oral hygiene (owed to poor dexterity), and alcohol and tobacco use, risk factors that are detrimental to oral health.

 The strong correlation between several oral diseases and noncomunicable chronic diseases is primarily a result of the common risk factors [6]. Many general diseases have oral manifestations that increase the risk of the oral disease, which, in turn, is a risk factor for some systemic diseases. 

Among the institutionalized people, especially in the elderly, high prevalence of co–morbidities and barriers to care are observed, together with oral health challenges in relation to changing dentition status, caries prevalence, periodontal status, oral hygiene, edentulousness, limited oral functioning, denture related conditions, ill fitting removable dentures, oral cancer, xerostomia, pain and discomfort [8]. The wider meaning of oral health does not diminish the relevance of the two globally leading diseases: dental caries and periodontitis. Both can be effectively prevented through a combination of community, professional and individual action. An oral examination can detect a number of general diseases, including nutritional deficiencies, microbial infections, immune disorders, injuries or oral cancer. Salivary glands are a model of the exocrine glands and an analysis of saliva can provide important clues to general health.

One of the Healthy People 2020 Objectives is to increase the proportion of local health departments and community–based health centers, including homeless centers that have an oral health component. Institutionalized and homebound elderly have poorer oral health status than the active elderly. Life style factors significantly contribute to the disparities. Behavioral risk factors (oral hygiene, diet, tobacco use) are modifiable and there are positive experiences from intervention programs instrumental to improved oral health status of older people [3,8]. Oral health care is not adequately considered in most protocols on personal hygiene and general health for the care of home residents, which constitutes a high–risk group with respect to oral and overall health, because of infrequent check–ups, irregular eating habits, poor hygiene, general self–neglected and the side effects of medications. Providing preventive care to underserved population is a well–recognized challenge due to some barriers: cost of health services, limited availability, difficult access and patient reluctance [9]. 

## Meth

The center for the homeless in Bucharest, where the study was conducted, functions as a night and daytime shelter and offers medical care; it shelters aged persons, people with disabilities, unemployed people who do not own a home and young people (who previously lived in orphanages until they were 18 years old, and then became homeless). There was no dental office within the center. Entrants undergo compulsory medical examinations, but are not orally examined before institutionalization or while they live in the center. 

The ethical committee of ‘Carol Davila’ University of Medicine and Pharmacy in Bucharest and the manager of the center approved that the study should take place and all but four of the residents consented to take part in it. The study group consisted of 51 adults from a community that is disadvantaged from a socio–economic and medical point of view: frequently with multiple general diseases, chronic conditions treated with medicines that produce oral side effects, a high need of treatment for the general and oral diseases and the lack of a dental office. 

The dental examinations were undertaken by using the procedures recommended by the World Health Organization (WHO, 1997). The decayed, missing and filled teeth (DMFT) score was used for the assessment of the dental status. The subjects were orally examined from a clinical point of view, in order to detect any oral abnormal mucosal conditions and the presence of oral dryness. The evidence of dry lips, dryness of buccal mucosa and the lack of saliva upon palpation was accepted as determinants of dryness [10]. Then, the subjects received a questionnaire, with regard to the presence of the risk factors for the general and oral health. The oral hygiene level was assessed by using the plaque index described by Silness and Löe (score range 0–3). General health status and drug treatments of subjects were evaluated based on medical records. Stata 11C statistical software (StataCorp LP, Texas, USA, version 2009) was used for data analysis.

## Results

The mean age of all subjects was 58 years old, with an age range between 22 and 84 years old. The gender distribution was approximately equal: 24 females and 27 males.

The exposure of the subjects to common risk factors for oral and general health (tobacco use, alcohol consumption, diet, history of cancer) is presented in Table 1. 

**Table 1:** Characteristics of the study population

Characteristic of the subjects	n	Male	Female
Age (in years)			
18–34	7	5	2
35–44	2	1	1
45–64	19	13	6
>65	23	8	15
Tobacco use			
Never	17	7	10
Former	2	0	2
<10 cigarettes per day	16	8	8
>10 cigarettes per day	16	10	6
Alcohol consumption			
No	32	21	11
Yes	19	6	13
In the last year	25	16	9
In the last month	7	5	2
Last dental visit			
6 month–1 year	2	2	0
Many years	40	21	19
Never	9	5	4

Thirty–four participants reported that they were or had been smokers, 29 claimed to smoke daily, and the others occasionally. 52% of the subjects declared the alcohol consumption; the value might be lower than real, since, although the subjects were explained the purpose of the study and were assured of anonymity, some might have hidden alcohol (a condition to remain in the center is not to consume alcohol) consumption. The center menu provides the diet (fruit and vegetable consumption) of subjects; in the past, the situation was as it follows: 41.7% preferred a diet rich in fruits, and vegetables were consumed by 31.4%. Six subjects reported other risk factors such as history of cancer (lateral–cervical, rectal cancer and pancreatic cancer) and family history of cancer (cerebral, stomach and breast cancer). 

*Oral health assessment*: the DMFT was 15.63 (SD 9.51) and largely associated with missing teeth (78.8%).  Decayed teeth were found in 62.4% of all subjects and 15 institutionalized persons (29.4%) were caries free. The average number of teeth with caries was 3.52 per person; the number of the teeth extracted was 12.3 per person and there were no teeth with fillings. Among dentate subjects, the mean number of teeth was 16.7. The oral hygiene level is poor, with high levels of dental plaque; the score for Silness and Löe index is 2.3. No lesions of the oral mucosa were detected with malignant potential or suspected oral cancer. 

The main aspects of oral health status, including normative dental treatment need are presented in Table 2. 

**Table 2:** Oral health status

Variables	n	%
Number of remaining teeth		
Group 1 (20 or more)	24	47.0
Group 2 (with 10 to 19)	8	15.6
Group 3 (with 1–9)	16	31.3
Group 4 (edentulos)	3	5.8
Caries prevalence	32	62.4	
Dentures status		
No denture	2	3.9
Denture worn	1	1.9
Crowns and fixed restoration	5	9.8
Prosthetic treatment need		
No denture /dentures need	1	1.9
Fixed restauration need	23	45.0
Removable dentures need	17	33.3

The use of only clinical indicators for oral health status and treatment that needs evaluation is recognized to have serious limitations; health is no longer defined in terms of illness and disease, and socio–dental indicators that assess physical, psychological, and social aspects of well–being must also be considered in further studies [11]. Poor oral health increases the risks of general health, and compromised chewing and eating abilities affect the nutritional intake.

*General health assessment*: systemic diseases affect oral health and vice versa. Subjects show many systemic diseases (cardiovascular 50.9%, rheumatoid 35.2%, metabolic 33.3%, digestive 39.2%, mental 33.3%, neurological 24.2%, diabetes 17.8%). Most of the subjects were on medications with an average of two (mean 2.21, SD 1.10) and the maximum number per person was six. They received general medications including: antidepressants/antipsyhotics (41.1%), antihypertensives (39.2%), antifungal (33.3%), vasodilators (33.3%), diuretics (19.6%), calcium antagonists (9.8%), antiepileptics (7.8%), as shown inTable 3. The high prevalence of multi–medication therapies may further complicate the impact on oral health. Several medications (especially psychotropic medication, the most prevalent in this study) have a negative effect on oral cavity of subjects by inducing xerostomia (29.4% of subjects complained), a condition with a severe impact on oral health: it increases the risk of dental caries, periodontal disease and oral infections (such as glossitis, stomatitis and yeast infections), fissuring of the corners of the mouth and lips and difficulty in chewing, speaking and swallowing; others drugs (calcium antagonists and antiepileptics) can adversely affect gingival tissues (overgrowth not found in the present study). 

## Discussion

Subjects included in this study present a series of general conditions that must be considered along with oral health status in the assessment of normative treatment needs. This cross–sectional study can be considered a pilot study for the future one, with a larger representative sample.

The majority of the subjects are exposed to common risk factors for oral and general health. Dental treatment needs are great, but it is not possible to meet them because there is no dental practice in the center. The need to involve decision makers in creating the organizational framework for the establishment of dental offices in asylums for the elderly and homeless centers emerges; the dentists must engage in regular check–ups and as much as possible in dental treatment. 

Because oral and other chronic diseases have determinants in common, more emphasis should be on the common risk factor approach, in order to improve health conditions in general for whole population and for groups at high risk, thereby reducing social inequities. 

By integrating oral health into strategies of promoting general health and by assessing treatment needs in sociodental ways, health planners can greatly enhance both general and oral health. Oral and general diseases can be effectively prevented through a combination of community, professional and individual action. It is important to reduce the burden of diseases and disability, especially in poor and marginalized populations, as well as to promote healthy lifestyles and to decrease risk factors that arise from environmental, economic, social and behavioral causes.

**Table 3:** General health status

Systemic diseases	n(%)	Medications	n(%)
Cardiovascular	26 (50.9)	Diuretics	10 (19.6)
Cerebrovascular	7 (13.7)	Antiarrhythmics	4(7.8)
Metabolic	17 (33.3)	Antihypertensives	20 (39.2)
Diabetes	9 (17.8)	Calcium antagonists	5(9.8)
Rheumatoid	18 (35.2)	Antiepileptics	4(7.8)
Digestive	20 (39.2)	Antidepressants/antipsyhotic	21 (41.1)
Mental	17 (33.3)	Vasodilators	17 (33.3)
Epilepsy	2(3.9)	Platelet antiaggregants	11 (21.5)
Neurological	20 (24.2)	Antifungal	17 (33.3)
Respiratory	6 (11.7)	Iron	8 (15.7)
Anemia	6 (11.7)	NSAIDs	10 (19.6)
Iron chronic deficit	8 (15.6)	Vitamins	13 (25.5)
